# Polypropylene Modified with Carbon Nanomaterials: Structure, Properties and Application (A Review)

**DOI:** 10.3390/polym17040517

**Published:** 2025-02-17

**Authors:** Lusine Elbakyan, Irina Zaporotskova

**Affiliations:** Institute of Priority Technologies, Volgograd State University, 100 Prospect Universitetsky, Volgograd 400062, Russia; irinazaporotskova@gmail.com

**Keywords:** polypropylene, carbon nanotubes, polymer nanocomposite, nanoparticles, electronic and mechanical properties

## Abstract

Polymer materials are increasingly used in all spheres of human activity. Today, it is difficult to imagine our life without the use of polymer products. Polymers have played a crucial role in the development of many industries and, of course, can be considered as one of the main drivers of technological progress. The research on the creation of new polymer materials that are obtained by modifying known polymers with various fillers, including nanomaterials, is widespread nowadays. In the foreseeable future, the time will come for modified polymer composites, when up to 75% of all things and materials that surround us will contain nano-additives. Due to their unique properties, these polymer compounds are in demand not only in industry and in everyday life, but also in medicine. One well-known nanomaterial is carbon nanotubes. The existing applications of nanotubes are almost limitless. Using them as modifying additives, it is possible to improve the properties of almost all known materials: polymers, alloys, plastics, rubbers, concretes, etc. In this review paper, the well-known polymer polypropylene and carbon nanotubes are selected as the main subjects of this study. This choice is due to their high demand in medicine, electronics, construction, etc.

## 1. Introduction

Polymers are versatile materials with many unique properties, such as low density, strength, flexibility, easy machinability, etc. Despite the many advantages of these materials, their mechanical properties may limit the use of polymers in some engineering fields. One of the most pressing issue is the possibility of obtaining polymer materials with improved properties. Thus, various types of polymers were used to manufacture new materials with unique properties. With the development of technology, there was a need to obtain more advanced polymers. Composites reinforced with fine fibers or particles have been developed. Silicon dioxide, technical carbon, metal particles, etc., were usually used as fillers. In order to achieve the desired characteristics, a high filler content was required, so the cost increased and machinability became more difficult. Nanofillers were used to achieve improved properties with a lower filler content. Polymer nanocomposites are a new type of composite materials that have received special attention in both research and industry [1]. Various types of nanoparticles, such as carbon nanotubes, graphene, nanocellulose, halloysite and other nanoparticles have been used to obtain nanocomposites with different polymers. Polymer nanocomposites offer excellent opportunities for exploring new functionalities not typically found in conventional polymers. The field of nanocomposites is one of the most promising and developing areas of research [2].

There are many polymers that scientists have studied with various nanofillers. For example, such polymers include polyethylene [3], polyacrylonitrile [4,5,6], polymethylmethacrylate [7], polyvinyl alcohol [8], polyethylene terephthalate [9,10] and other polymers.

Recently, researchers have been actively working on the creation of composite polymer materials reinforced with various nanomaterials, including carbon nanotubes (CNTs) [11,12,13,14,15,16]. The analysis of a large number of scientific papers [16,17,18] has shown that carbon nanotubes, due to their outstanding thermal, mechanical, electrical and optical properties, hold a special place in the field of composites. The ever-growing interest in the inclusion of CNTs in various matrices has stimulated efforts to manufacture polymer nanocomposites based on CNTs and polymer/fiber nanocomposites with exceptional characteristics [11]. In addition, the development of such materials stands out as one of the most significant achievements for nanoscience, biomedical [19,20,21], aerospace, civil [22] and other industries [23,24]. The choice of a specific polymer matrix depends on the intended application and the desired combination of properties of the modified material. Polymers include acrylonitrile butadiene styrene (ABS), polyvinyl chloride (PVC), polymethylmethacrylate (PMMA), polyethylene terephthalate (PET), polylactic acid (PLA), polyvinylidene fluoride (PVDF), polyacrylonitrile (PAN), polypropylene (PP), natural rubber (NR), polyethersulfone (PES), polyurethane, polyimides and epoxy resins [16]. When CNT is added to these polymers, the resulting material becomes stronger, tougher, more durable, and improves thermal and electrical conductivity. Researchers are constantly experimenting with new combinations of polymers and CNTs to create materials with specific properties for various industries.

In this review paper, the well-known polymer polypropylene and carbon nanotubes are selected as the main subjects of this study. This choice is due to their high demand in medicine, electronics, construction, etc.

## 2. Polypropylene

**Figure 1:**
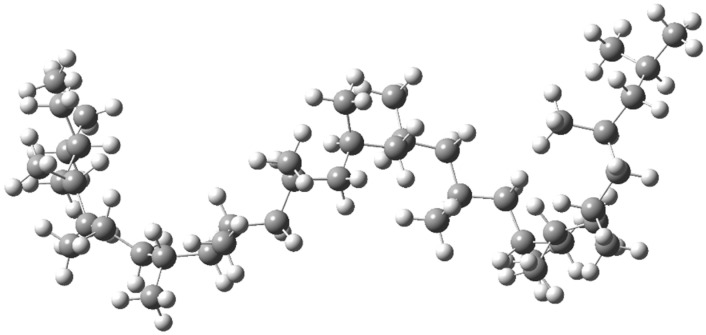
The propylene molecule.

Polypropylene (PP) is a chemical compound related to synthetic polymers [25]. It is a product of polymerization of propylene and belongs to the class of polyolefins (Figure 1). It is a durable, rigid plastic that is produced with different molecular weights and crystallinities [26].

This material is valued in many sectors of the economy due to its physico-chemical properties. From the point of view of industrial production, one of the most demanded properties of this substance is its high chemical resistance to various types of compounds and substances. In particular, polypropylene is extremely neutral against chemically aggressive acids, bases and solvents. On the other hand, it is not resistant to non-polar liquids, including benzene, methyl chloride or carbon tetrachloride. Another property of polypropylene is its low vapor permeability. In addition to its good insulation properties, this material also has high air permeability and no water absorption. This versatile polymer is used in a wide range of applications, from fibers and flat filaments used in industrial bags and outdoor carpets to packaging films, household appliances, electrical and medical devices, and automotive parts [27].

Today, polypropylene has an important place in the global production of synthetic plastics. Its wide application in various industries such as packaging, automotive industry, medicine, textiles, building materials, etc., contributes to the growth of demand. The expected increase in polypropylene production to 88 million tons by 2026 reflects the upward trend in plastic consumption, as well as the development of recycling technologies and improvements in its properties.

### 2.1. Composites of PP with Various Nanoparticles

Various fillers are widely used to improve the properties of polypropylene. Nanotechnology has been used to improve the properties of PP, mainly by testing pure resin and several nanofillers. For example, in [28], the hybrid reinforced composites with talc fillers and cellulose nanofibers were proposed as a new method for manufacturing fused filaments for polypropylene. It has been found that hybrid reinforcement allows polypropylene composites to achieve a maximum voltage of 1.45 times higher than that of pure polypropylene. Depending on the type of filler, mechanical [29,30,31,32,33], rheological [30,33,34], thermal [30,32,35,36] and electrical [36,37] properties can be improved.

Thus, in [30], the effectiveness of using titanium nitride (TiN) as a reinforcing component was studied in order to produce a new generation of printed biocomposite nanofibers based on polypropylene. It has been established that the mechanical reaction of a nanocomposite with 2.0 wt.% titanium nitride (TiN) is resistant to wear, corrosion and erosion. Due to these characteristics, titanium nitride is in great demand in areas where strength and resistance to mechanical damage are important. The addition of nitride nanoparticles to polymer matrices can improve the mechanical and thermal characteristics of the matrices, expanding the scope of their potential applications.

The raw materials underwent a drying procedure at 60 °C for 24 h before the creation of the nanocomposites to eliminate any remaining moisture. For the initial dispersion of TiN nanoparticles in a polypropylene polymer, a blender (4000 revolutions per minute) was used for 30 min at 23 °C. After the mixing stage, the mixtures underwent another drying cycle. Next, the resulting mixture was converted into filaments using an extruder. Then, using a shredder, these threads were crushed into granules, which underwent additional processing. To successfully combine the polypropylene matrix with TiN particles and obtain filaments, an extruder with a special screw was used for mixing raw materials and certain conditions. The study used a two-stage extrusion process involving the sequential use of two different extruders. This methodology was aimed at subjecting nanocomposites to additional thermomechanical mixing processes in extruders in order to achieve optimal particle dispersion in the matrix. The concentration of filler showed improvements in the properties of the material in all experiments, including tensile strength (41.5%), bending (33.7%) and impact strength (18.0%). Microhardness improved by 44.8% for a nanocomposite with a filler concentration of 2.0 wt.%. Overall, TiN’s effectiveness as a reinforcing component in 3D printing was proven, making the proposed nanocomposites a good solution when improved performance is required. Hadi NJ and Mohamed DJ [34] studied the effect of inclusion of silicon nanoparticles on the rheological properties of polypropylene after its processing. Their results show that the melt flow rate increases with increasing nanoparticle content. In [35], the authors studied the effect of CaCO_3_ nanofillers on the crystallinity and thermal conductivity of primary polypropylene and polypropylene waste. It was found that crystallinity increases with an increase in the CaCO_3_ content. This is due to the fact that CaCO_3_ nanoparticles fill voids and orient the chain in such a way that the degree of crystallinity increases. They also found that the values of thermal conductivity increase with an increasing concentration of nanoparticles and level of crystallinity.

Vidakis and other authors in [33] investigated rheological and mechanical properties such as flexural and tensile strength, as well as the modulus of elasticity of new materials based on polypropylene (PP) doped with ceramic nanoparticles of silicon nitride (Si_3_N_4_). The authors point to the extraordinary qualities of silicon nitride (Si_3_N_4_) ceramics, including their strength, great durability and resistance to cracks, as well as their capacity to withstand sudden temperature changes. Si_3_N_4_ is a biological material and is commonly used in medical sectors. Two extrusion operations were performed to improve the distribution of Si_3_N_4_ nanoparticles in the polymer material. The method is similar to the method described in [30]. The study proved the ability of Si_3_N_4_ ceramic nanoparticles to improve the mechanical characteristics of PP-based compounds and products produced by 3D printing. Thus, the best results were obtained at a filler concentration of 2% by weight (a tensile modulus of elasticity of 21%, a bending strength of 15.7% and high values of the other evaluated properties).

In addition, the use of nano-additives can improve the resistance of polypropylene to processing, reducing the likelihood of thermal decomposition and increasing its stability during processing [38,39,40]. Thus, in [38], the effect of processing on the properties of polypropylene filled with CNT was studied. The chemical structure, melt rheology, mechanical properties and morphology were characterized using recycled materials. Both the pure and filled materials showed a decrease in viscosity during processing, but the changes were more pronounced for pure materials. Young’s modulus, yield strain and stress for both pure PP and PP filled with CNT proved to be minimally affected by recycling. Deformation and tensile stress for pure PP decreased during processing, but only minor changes were found for PP filled with CNT. PP filled with CNT showed an increase in strength during processing due to changes in crystallization behavior. Abhishek Kumar Pathak and Tomohiro Yokozeki in [39] established that carbon nanofibers (CNFs) as fillers play an essential role in maintaining the mechanical and thermal properties of recycled PP nanocomposites. Polypropylene was separately mixed with CNF (30 wt.%) in a biaxial mixing extruder. A concentration of 30 wt. CNF/PP-CNF nanocomposites was manufactured using injection molding at a temperature of 200 °C. No impregnating agent was used in the manufacturing of the nanocomposite. The next sample, a PP/CNF nanocomposite, was processed in a biaxial mixing extruder, and new PP/CNF plates were obtained. The recycled PP/CNF nanocomposite was made by injection molding under the same conditions as the first one. The second recycled PP/CNF nanocomposite was obtained in a similar way. The authors propose using the recycled PP-CNF nanocomposite for the manufacturing of automotive parts due to the low change in mechanical and thermal properties, as well as its stability for other structural applications. Article [41] provides an overview of the use of various nanofillers, such as clay, calcium carbonate (CaCO_3_), silicon dioxide (SiO_2_), zinc oxide (ZnO), carbon black (CB), carbon nanotubes (CNT), antioxidants and other nanofillers, in the recycled matrix PP. For example, the mechanism for producing PP/SiO_2_ composite material consists of several stages. First, the polypropylene sample is cut into granules. SiO_2_ NPs are immersed in alcohol solution and mechanically mixed. The mixing of PP and SiO_2_ is performed using a twin-screw extruder under certain operating conditions of the extruder. Due to their fractal structure and the high specific area, fumed silica is subject to self-aggregation and can consequently form a network of connected particles in the molten polymer, promoting significant changes in physical properties such as rheological parameters. Thanate Ratanawilai and Nakanat Raksarak, in their study [42], suggested using fibers of plant origin as reinforcing fillers in recycled polypropylene, namely, fibers from coconut, oil palm, and corn plants (specifically, coconut (*Cocos nucifera*), oil palm (*Elaeis guineensis* Jacq.) and corn (*Zea mays*)). Nanocomposite films based on post-consumer recycled polypropylene pellets and nanosilica were obtained by extrusion. Virgin polypropylene was used as a commercial reference for polypropylene in the production of bioriented extrusion or co-extrusion films. The films were produced in a twin-screw extruder with a temperature profile ranging from 180 °C to 195 °C from the feed to the extrusion head. The screw speed was 35 rpm, and the torque was from 40% to 50%. Before extrusion, the polymer and filler were dried at 100 °C for 24 h, and then manually mixed until a homogeneous mass was obtained. The authors found that the choice of different fibers significantly affected the overall mechanical, thermal, and fire-resistant characteristics of the samples. The increased fiber content in the samples had a negative effect on their flammability and had no effect on the thermal stability of the samples. However, the overall mechanical properties of the samples were improved, with the exception of flexural strength. At the same time, the samples containing coconut fiber showed significantly higher mechanical and physical properties. Eliezer Velásquez and his group, in [43], considered the possibility of increasing the value of recycled flexible polypropylene after consumption (post-consumer recycled flexible polypropylene PCPP) by including colloidal nanosilicon (nanosilica NS) in it. For this purpose, the effect of concentration and type (hydrophilic and hydrophobic) of NS on the morphological, mechanical, sealing, barrier and overall migration properties of PCPP films was studied. Due to their fractal structure and the high specific area, NS is subject to self-aggregation and can consequently form a network of connected particles in the molten polymer, promoting significant changes in physical properties such as rheological parameters. It was found that the inclusion of NS leads to an improvement in Young’s modulus and tensile strength at 0.5% by weight and 1% by weight. At the same time, NS in a concentration of 1 wt.% did not affect the permeability of films to water vapor and oxygen. Thus, studies have shown that with an NS content of 1% by weight in the PCPP polymer matrix, an improvement in the overall characteristics of the studied packaging properties is observed. In [44], electrically conductive PP composites were obtained using the melting–compounding method in a twin-screw extruder by adding fillers in the form of multi-walled carbon nanotubes (MWCNTs), carbon black CB and expanded graphite EG. The optimal composition of fillers was found, namely MWCNTs (4 wt.%), CB (5 wt.%) and EG (30 wt.%), at which point the polymer material acquires an electrical conductivity of 39.6 S/cm and a bending strength of 29.4 Mpa. This comprehensive experimental study offers potential recommendations for the production of electrically conductive thermoplastic composites for the manufacturing of bipolar fuel cell plates.

Table 1 shows the types of nanofillers and the characteristics that are improved in polypropylene due to these nanofillers. The third column of the table provides a link to the references.

### 2.2. Composites of PP with Single- and Multi-Walled Carbon Nanotubes

There is a significant amount of scientific work that has proven that carbon nanotubes (CNTs) occupy an prominent position in the field of composites and nanomaterials and are widely used in many industries. Over the past decade, CNTs have been considered as promising nanoscale reinforcing fillers with random orientation, similar to conventional composites reinforced with short fibers [45,46,47]. Carbon nanotubes have a whole set of properties that are of interest in the manufacture of polymer composites with a new or improved set of properties. These include strength properties, thermal and electrical conductivity, optical, magnetic, sorption and other characteristics [7,48,49]. The ever-growing interest in the incorporation of CNTs into various matrices has stimulated ongoing efforts to manufacture CNT-based nanocomposites. In addition, the development of new CNT-reinforced polymers (engineering and natural) and polymer/fiber nanocomposites with exceptional characteristics stands out as one of the most significant achievements in materials science, nanoscience and thebiomedical and aviation industries [11].

CNTs are considered to be a material with record-high tensile strength (≈60 GPa) and Young’s modulus (≈1 TPa) values, which is determined by the strong chemical sp2 bond between the carbon atoms composing the nanotube. According to the quantum chemical calculations of the Young’s module, it was found that CNTs with a larger diameter are more durable (E = 1.2 TPa) than CNTs with a smaller diameter (E = 0.76 TPa); CNTs with the “armchair” configuration are more durable (E = 1.2 TPa) than tubes with the same diameter, but with the “zig-zag” configuration (E = 0.825 TPa) [50].

**Figure 2:**
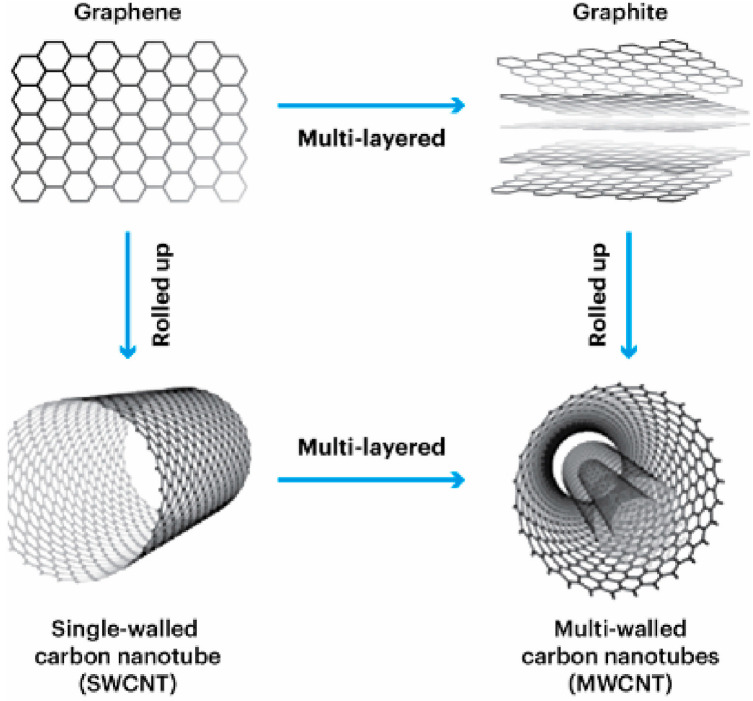
SWCNT and MWCNT structures [16].

When discussing the electrical properties of CNTs, one should distinguish between the conductivity of individual nanotubes—single-walled CNTs (SWCNTs) or multi-walled CNTs (MWCNTs) (Figure 2); the conductivity of a CNT array; and the conductivity of a material in which nanotubes are isotropically or anisotropically distributed. It has been established that the electrical conductivity of an isolated CNT depends on chirality, the presence of structural defects, covalently attached radicals (OH, CO, etc.) and other factors. For example, achiral “armchair”-type SWCNTs have metallic conductivity, while “zigzag”-type SWCNTs have semiconductor conductivity. Under the condition of low defectiveness, the conductivity of metallic CNTs is about 10^9^ A/cm^2^. For comparison, a copper wire can withstand a current density of no more than 10^6^ A/cm^2^ and melts at a higher current density. Chiral SWCNTs can also have either semiconductor or metallic conductivity, depending on the magnitude of the chiral angle θ and diameter d. In isolated MWCNTs, neighboring cylindrical layers have different chirality, which changes randomly during the transition from one layer to another. Due to the relatively weak interaction between the individual graphene cylinders embedded in each other (the distance between the individual cylinders corresponds to the interplane distance in graphite), electric current flows predominantly through the wall of the outer tube. In general, the electrical conductivity of MWCNTs and SWCNTs may vary. The electrical conductivity of the CNT material largely depends on the degree of contact between adjacent tubes, as well as on the presence and composition of impurities. Finally, the conductivity of the material in which the nanotubes are distributed depends on their content in the dispersion medium. The introduction of a small amount (up to 1% by weight) of electrically conductive nanotubes with a high aspect ratio into a dielectric matrix results in its electrically conductive properties. This is due to the formation of a single electrically conductive network in the structure of the material [51]. Among the thermal properties of CNTs, there are, in particular, characteristics such as thermal conductivity, heat capacity and the coefficient of thermal expansion. It has been established that CNTs have a thermal conductivity greater than that of diamond or any other material of natural origin. Thus, according to literature data, the coefficient of thermal conductivity along the CNT axis of various structures (length, diameter) is mainly from 500 to 5500 W/mK (in particular, CNT 175–5800 W/mK, CNT > 3000 W/mK). For comparison, the thermal conductivity of silicon is 150 W/mK and that of copper is 400 W/mK. At the same time, the coefficient of thermal expansion of CNTs is lower than that of copper. Such features of the structure and thermal properties of CNTs are attractive for use as components of electronic devices (fast and efficient removal of excess heat from the internal parts of semiconductor chips) or as components of composite materials [52].

**Figure 3:**
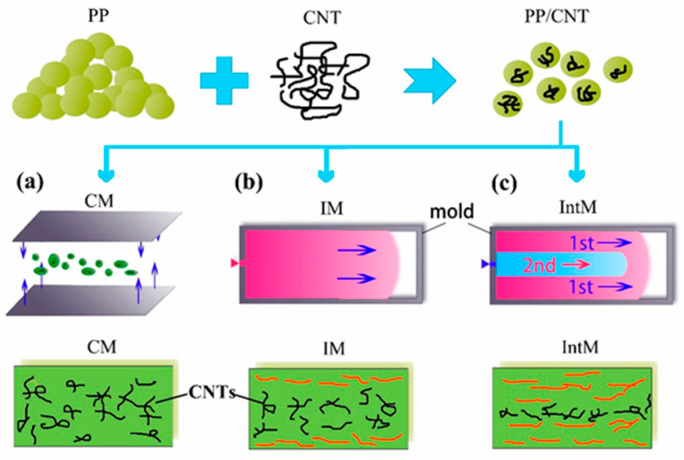
Schematic of molding methods and CNTs orientation: (**a**) compression molding (CM); (**b**) conventional injection molding (IM); (**c**) intermittent injection molding (IntM) [60].

It is known that the main problem in obtaining composite materials is to ensure the uniform dispersion of nanofillers in a polymer matrix. This largely determines the properties of the composite materials obtained. There are many studies that provide methods for dispersing CNT particles for the manufacturing of polymer composites [53,54,55,56,57,58]. For example, ref. [53] describes a method for manufacturing electrically conductive polymer composites based on polypropylene using a new physico-mechanical technology. To improve the dispersion of CNT particles, a combination of dispersing agents such as superplasticizer and silica dust, ultrasonic treatment and solvent-based melting treatment were used in this technology. Polypropylene and xylene were used as a binder and solvent, respectively. The test results showed that the proposed technique significantly improves the dispersion of carbon nanotubes. In addition, the electrical resistivity of polypropylene composites made using the proposed method was quite low. In [59], thermoplastic polypropylene was introduced into a mixer (made by Brabender). Then, after melting the polymer matrix, CNTs with a certain operating mode were introduced. The rotation speed was 30 revolutions per minute, the temperature of the mixing chamber was 180 °C and the mixing time was 30 min. Dashan and his colleagues [60] proved the influence of orientation and dispersion on the electrical conductivity and mechanical properties of the CNT/PP composite. Composite materials with different CNT contents were mixed using a twin-screw extruder. Three molding methods were then used to produce different shear stresses, such as compression molding (CM), injection molding (IM) and interval injection molding (IntM). Then, three molding methods were used to provide different shear stress, such as compression molding (CM), injection molding (IM) and intermittent injection molding (IntM) (Figure 3).

The authors point to various changes in the molecular orientation with increasing CNT content, depending on the chosen method. It was concluded that a high orientation leads to a high tensile strength and modulus of elasticity, respectively, improving mechanical properties. At the same time, the high orientation and high dispersion of CNTs prevent the formation of a conductive mesh. In [61], the effect of ultrasound on the chemical structure of polypropylene and its composites with different CNT contents (0%, 1%, 3% and 5% by weight) was studied. The composites were extruded using a traditional single-screw extruder and immediately irradiated in a static mixer. The results showed that ultrasonic irradiation caused a slight oxidation of the polypropylene structure and a 13% decrease in its molecular weight. As a result of ultrasonic irradiation, an increase in the crystallinity of polypropylene was also observed, which is explained by the improved nucleation effect of nanotubes. The ultrasonically treated PP/MWCNT composites showed a better dispersion of nanotubes in a polypropylene matrix, which led to a 30% increase in the elastic modulus, a 45 °C increase in thermal decomposition temperature, an 11-order-of-magnitude increase in volumetric resistivity, and a 25% increase in thermal conductivity. In addition, the SEM results showed that the structure of the MWCNT was preserved during processing due to the low shear stresses created by the single-screw extruder, while maintaining dispersion using an ultrasonic static mixer.

Table 2 below provides the main methods of obtaining PP/CNT composite materials, which were described in the article above. The table also contains the information about the properties that the authors investigated and the literature sources consulted.

### 2.3. The Use of CNTs in a PP Matrix to Improve the Mechanical and Thermal Characteristics of a Polymer Material

**Figure 4:**
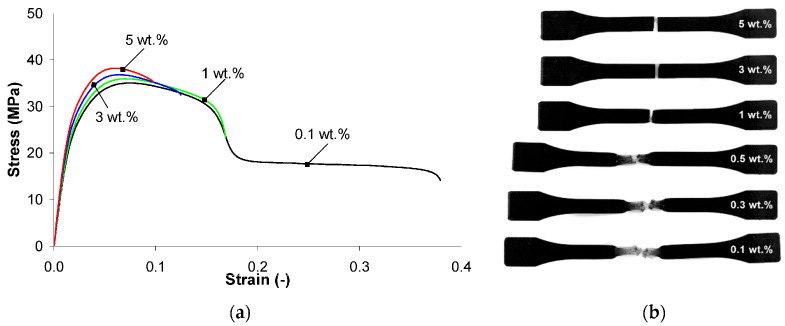
Stress-strain curves for the PP/MWCNT nanocomposites: (**a**) at 200 °C and 100 mm/min; (**b**) injection-molded samples after testing [62].

**Figure 5:**
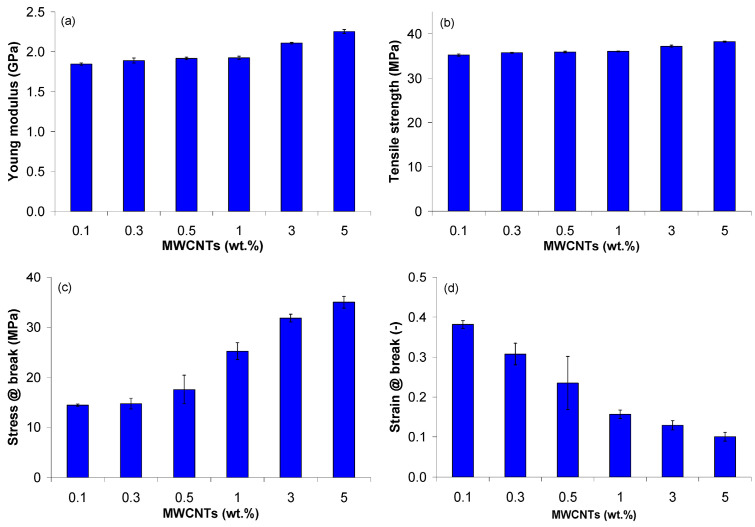
Mechanical properties of the PP/MWCNT nanocomposites: (**a**) Young modulus; (**b**) tensile strength; (**c**) stress at break; (**d**) strain at break at 100 mm/min [62].

In [62], a comprehensive study was conducted to study the effect of MWCNTs on the physical, thermal, mechanical and electrical properties of PP/MWCNT nanocomposites. The experimental results were supplemented by theoretical calculations of melt shear viscosity (Cross model) and specific volume (2-domain Tait model). It was found that the presence of MWCNTs does not significantly affect properties such as melting and crystallization temperatures or the degree of crystallinity of PP/MWCNT nanocomposites. When adding up to 5 wt.%, the PP/MWCNT nanocomposite is still a non-Newtonian liquid, and its shear liquefaction properties at high shear rates make nanocomposites suitable for processing by extrusion and injection molding. The specific volume of PP/MWCNT nanocomposites decreases with increasing MWCNT content, especially in the range of 1–5% by weight, which leads to better dimensional stability after processing in the melt. The thermal conductivity of the PP/MWCNT nanocomposite increases with the growth of MWCNT. The highest value of thermal conductivity (0.35 W/m·K) was achieved in the solid state, at 5 wt.% MWCNT. It was experimentally observed that the tensile modulus, tensile strength and tensile stress gradually increase with increasing the MWCNT content. In addition, elongation at break decreases significantly with increasing the MWCNT content. The authors also conducted theoretical studies within the framework of the Cross model and the modified two-domain Tate model, which successfully predicted the melt shear viscosity and specific volume as a function of the number of multi-walled carbon nanotubes (MWCNTs). To summarize, data on the properties of materials, supplemented by Cross and Tate models, can be used for the numerical modeling of production processes such as injection molding and extrusion to predict the behavior of PP/MWCNT nanocomposites at an early design stage (Figure 4 and Figure 5).

**Figure 6:**
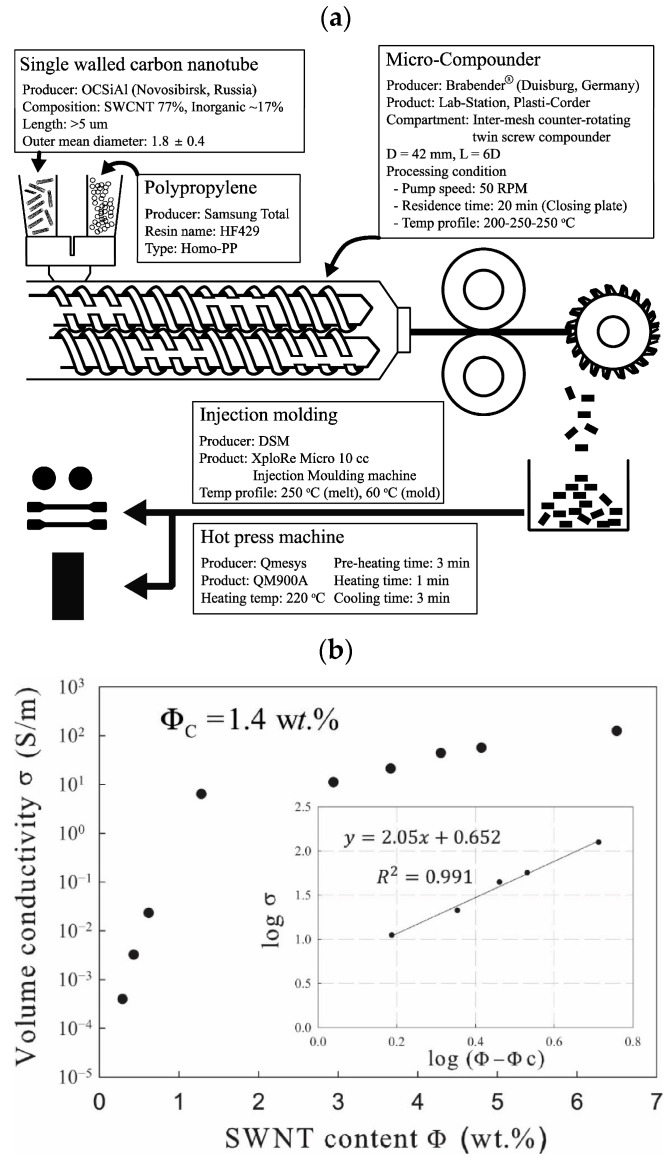
(**a**) Schematic diagram of the sample preparation of the PP/SWNT composite for this study; (**b**) electrical conductivity as a function of SWNT content [63].

In [63], a melt mixing technique was used with the time-controlled dispersion of single-walled carbon nanotubes (CNTs) in a polypropylene matrix. The composites were prepared using a twin-screw extruder equipped with a return transportation element, with the addition of various amounts of SWCNTs, from 0.29 to 6.56 wt.%. During rheological measurements, it was found that the holding time for 20 min shows the lowest average molecular weight and low average molecular weight by weight, which leads to a low polydispersity index. At the same time, the rheological percolation threshold was reached at 0.29 wt.% SWCNTs. Morphological properties were evaluated using optical microscopy and FE-SEM. In all composites, the dispersed CNT agglomerates occupied less than 3% of the total area. Relatively large agglomerates with a size of more than 200 microns were found in composites containing more than 1.28 wt.% (Figure 6a,b).

**Figure 7:**
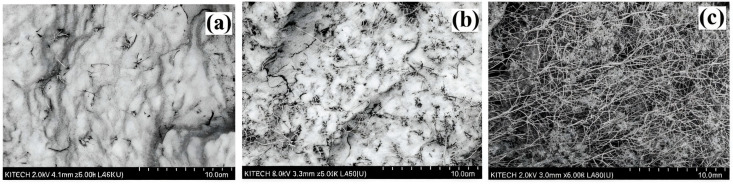
SEM images of PP/SWCNT composites with SWCNT: (**a**) 0.29 wt.%, (**b**) 1.28 wt.%, (**c**) 6.56 wt.% [63].

The decrease in the number of agglomerates from the larger diameter classes may indicate that this method may be effective in rupturing the large agglomerates for composites up to approximately 1.28 wt.% SWNT content. At low SWNT content (Figure 7a), most of the SWNTs are dispersed individually in a polypropylene matrix, and some are formed as small aggregates. As confirmed by the results of rheological property measurements, despite the small number of introduced CCTSs, the dispersed state of the CCTSs was good enough to transfer mechanical torque, which led to a rheological percolation threshold of 0.29 wt.% CCTS. As the SWNT content increases (Figure 7b), individual SWNTs gradually grow and begin to form SWNT networks, leading to small distances between particles. Figure 7c illustrates the SEM image of the highest quality PP/SWNT composite in this study. With such a high SWNTS load, the SWNT mesh dominates the properties of the PP/SWNT composite.

Despite the large agglomerates detected by morphological measurements under a high load of the CNT composite, the tensile strength and Young’s modulus increased linearly with increasing CNT content, which may mean that relatively large agglomerates were insufficient to cause stress cracking.

A number of scientific papers in the field of PP/CNT composite materials are related to the study of the effect of CNTs on the thermal properties of composite materials. In [64], the effect of the addition of carbon nanotubes (0.5 and 1.0 wt.%) on the crystallization process of isotactic polypropylene is analyzed. The study showed that the crystallization temperature increases with increasing nanotube content, while the crystallization of polymers does not change significantly. The critical cooling rate at which polypropylene does not crystallize increases with increasing carbon nanotube content. Using the critical cooling rate and nanotube content, a nucleation efficiency parameter was developed that does not depend on the crystallization temperature or the load on the CNTs. In [65], the mechanical and thermal properties of composite materials based on polypropylene reinforced with multi-walled carbon nanotubes (MWCNT) were studied. Samples with different MWCNT contents (0.4, 0.8, 1.2 and 1.5 wt.%) were produced using a compression molding machine. The results showed an improvement in mechanical properties, namely, the tensile strength increased by 62.80% at 1.2 wt.% MWCNT, and the impact strength increased by 82.14% and 12.44% at 1.5 wt.%. An improvement in thermal characteristics was also established. Thus, the glass transition temperature increases at low wt.%. The obtained samples were also examined using SEM. The images confirmed the uniform distribution of multi-walled carbon nanotubes in the polypropylene matrix. The work [59] focuses on the development and determination of the thermophysical characteristics of the CNT/PP nanocomposite. The results of the study showed that nanocomposite samples with 5% CNT by weight had improved mechanical properties, with an increase in tensile strength and Young’s modulus to 11 and 33%, respectively, compared to the initial samples. It was also found that the thermal stability of nanocomposites increased by more than 30 °C compared to the base samples with fire resistance. The study [66] examines the effect of multi-walled carbon nanotubes (MWCNTs) on the thermal characteristics of polypropylene materials. A twin-screw extruder and an injection molding method were used to produce composite materials. The author sought to find out the effect of different MWCNT concentrations and dispersion models by excluding the compatibilizer from the study. The results showed a slight increase in the peak crystallization temperatures of polypropylene under non-isothermal conditions with the addition of MWCNTs. It is important to note that the study revealed a significant “percolation threshold” at 0.5 wt.% MWCNTs. A further increase in the content of MWCNTs in the absence of a compatibilizer led to a significant improvement in physical properties. When this threshold is lowered, the increased interface area between polypropylene and MWCNT significantly increases the thermal stability of polypropylene. The study also showed reduced shrinkage of composite fibers compared to control fibers, with the heat-treated fibers showing a narrow melting peak at 170 °C. The author stated the importance of using the obtained results in the development of materials with improved physical characteristics, as they provide valuable information on the effect of MWCNTs on polypropylene nanocomposites.

**Figure 8:**
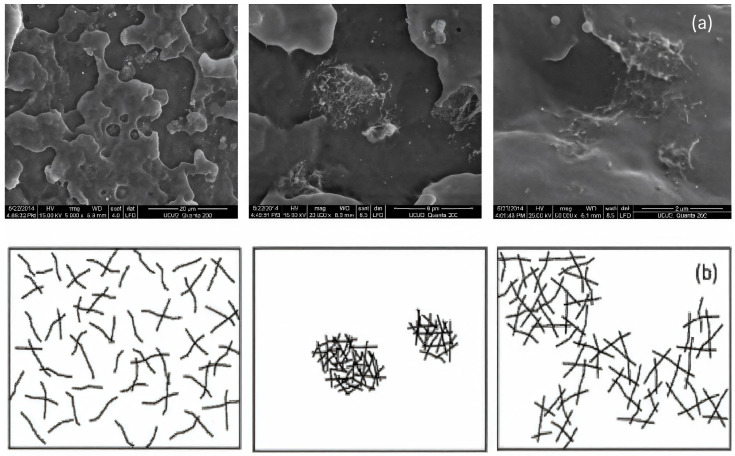
SEM images of the PP/MWCNT nanocomposite: (**a**) 5 wt.%; (**b**) schematic representation of MWCNTs [68].

There are also studies where the PP/CNT composite material is used as an additive to the base material, such as cement. In [67], the influence of multi-walled carbon nanotubes (MWCNTs) and polypropylene fiber (PP fiber) on the mechanical properties and durability of cement-based materials was studied, along with compressive and flexural strength, as well as impact strength. The results showed that the mechanical properties and durability of cement–based materials can be significantly improved with a 0.1–0.15% content of multi-walled carbon nanotubes. The compressive strength can be increased by 9.5%. Polypropylene fiber has little effect on the compressive strength of cement-based materials, but it significantly increases their impact strength. The content of polypropylene fibers in the amount of 0.2–0.3% is optimal for improving the mechanical properties and durability of cement-based materials (the mechanical properties and durability of the cement-based materials). The flexural strength increases by 19.1%, while the rate of dry shrinkage and the rate of water loss decrease by 14.3% and 16.1%, respectively. Studies have also shown that the resulting composite material has a higher resistance to freeze–thaw cycles. The three-dimensional mesh structure formed by polypropylene fiber in the composite material plays a role in hardening and cracking resistance, but has a certain negative effect on the porous structure of the composite material. The inclusion of multi-walled carbon nanotubes can improve the bonding properties (the bonding performance) of the polypropylene fiber and cement matrix, compensate for internal defects caused by polypropylene fiber and reduce the number of harmful voids and multiple harmful holes. Another important property of polymer materials is their viscoelastic behavior. For example, in [68], the authors suggested improving the viscoelastic properties of polypropylene materials by adding nanoparticles in the form of CNTs. A Micro Combi Tester (MCT3) with a Vickers diamond indenter tip was used to determine the mechanical characteristics. The presence of nanotubes in the PP matrix led to a change in the micromechanical properties of nanocomposites: both the indentation modulus and hardness steadily increased as the load on the nanotubes increased. However, the increase in indentation hardness was more significant with an increase in the CNT content from 1 to 5 wt. The hardness of the PP/MWCNT nanocomposite increased by about 43%, while the modulus of elasticity increased by only 28.5%. The authors also investigated the viscoelastic properties of the obtained composite material samples. It was found that the creep resistance of PP/MWCNT nanocomposites improved with the addition of MWCNTs, while creep decreased to 20% with an increase in MWCNTs from 1 to 5 wt. Figure 8a shows images of a PP/MWCNT nanocomposite with 5% MWCNTs by weight, obtained using a scanning electron microscope (three magnification levels). Studies have shown that the nanocomposite is homogeneous on the macroscale and heterogeneous on the microscale. Due to van der Waals interactions between CNTs, clustering and intertwining of MWCNTs (2a) were observed. It was noted that the size and density of agglomerates increased with increasing MWCNT content. Based on the images obtained using a scanning electron microscope, three types of distribution were identified, schematically shown in Figure 8b: (1) uniformly distributed MWCNTs (with an outer diameter of about 20–40 nm); (2) spherical aggregates/agglomerations of MWCNTs (up to 5 microns), turning into a homogeneous matrix; (3) loose agglomerates of MWCNTs. The authors indicate that the same morphology was observed in PP/MWCNT nanocomposites containing 1 and 3 wt.%.

However, despite the presence of agglomerates, the authors indicate that the addition of nanotubes to polypropylene has a positive effect on the mechanical properties of PP/MWCNT nanocomposites.

Attempts have also been made to improve the mechanical characteristics of polypropylene/carbon nanotube composites by additional treatment with microwaves [69]. Pawan Singh Bisht and al. studied the sensitivity to the rate of deformation of polypropylene nanocomposites treated with microwaves containing 10% of the volume fraction of multi-walled carbon nanotubes (MWCNTs). The use of microwave treatment ensures the uniform volumetric curing of composite components, which leads to increased mechanical strength. The study showed that the nanocomposite exhibits elastoplastic properties (maximum elongation of 1.10% at a 3 mm/min strain rate, maximum elongation of 1.10% at a strain rate of 3 mm/min). The ultimate strength increases from 5.74 MPa (at a strain rate of 1.52 mm/min) during uniaxial tensile testing to 13.8 Mpa (at a strain rate of 1 mm/min) and 15.7 MPa (at a strain rate of 2 mm/min). The maximum bending strength was a maximum flexural strength of 21.91 MPa, and the maximum bending modulus was a maximum flexural modulus of 2.69 GPa, at a deformation rate of 1.25 mm/min. However, the maximum elongation is observed at a deformation rate of 1.50 mm/min, which indicates a compromise between strength and ductility. This makes it possible to use such materials where the strength and performance of the material are crucial. In addition, the SEM study of nanocomposites with cracks reveals a random and spatial distribution of CNTs at low strain rates, while agglomeration and elongation of CNTs are observed at higher strain rates. This highlights the importance of understanding microstructural changes under different load conditions in order to optimize composite performance. There are a number of works where creep deformation and the reduction of polypropylene, as well as changes in these properties have been studied when carbon nanotubes are added to the polymer matrix [70,71,72]. In one of the recent papers on this topic [72], the authors propose increasing the creep resistance of the nanocomposite by doping carbon nanotubes into a polymer matrix of polypropylene. In this study, the creep properties of CNT/PP nanocomposite with different CNT content under constant loads at different levels were studied using atomic modeling with the coarse-grained MD modeling method [73], in which a cluster of atoms is transformed into a CG granule. In his article [74], Sabet investigated the effects of both single-walled and multi-walled carbon nanotubes (CNTs) on the thermal behavior and kinetics of crystallization of isotactic polypropylene (PP) composites. The inclusion of CNTs led to a noticeable increase in the crystallization temperature, without a significant change in the melting point of the polymer. In addition, the results revealed an increase in the critical cooling rate with an increase in the concentration of CNTs. A study on MWCNTs in PP nanocomposites revealed a key percolation threshold at 0.5% (by weight) MWCNTs. When this threshold was lowered, an improvement in physical properties was observed, without the need to use a compatibilizer. The increased interface area between polypropylene and MNT significantly increased the thermal stability of polypropylene. The results obtained make it possible to create new unique nanocomposite materials based on PP by doping with CNTs, which have improved thermal properties that are suitable for targeted applications requiring high productivity.

### 2.4. The Use of CNTs in a PP Matrix to Create Conductive Polymers

The prospects of using carbon nanotubes (CNTs) to impart high electrically conductive properties to the surface of materials have been proven [75,76,77,78,79]. Scientists around the world pay special attention to the possibility of creating conductive polymers and their potential applications. In recent years, many conductive polymers have been used to manufacture electronic devices, rechargeable batteries, artificial muscles, solar energy conversion and sensors [12,80]. In [81], a method for increasing the conductivity of a polypropylene/CNT nanocomposite was described by adding an external nucleating agent (NA) based on sorbitol and by controlling the cooling rate. Thus, the ability of CNTs to nucleate crystals in polypropylene (PP) was controlled. The authors obtained a slight increase in conductivity compared to PP/CNT during rapid cooling (~150 °C/min). However, during a slow cooling process (~1.5 °C/min), large PP crystallites were induced with only a small number of nuclei. Therefore, most CNT particles were unable to participate in the nucleation of PP crystals, leading them to concentrate and form conductive networks through an improved volume exclusion effect. This significantly increased the conductivity of PP/CNT nanocomposites, and the percolation threshold was significantly reduced from 0.75% by weight to 0.36% by weight. In this paper, the authors emphasize the crucial effect of controlling the nucleation ability of CNTs and the size of polymer crystallites on conductivity using slow cooling in addition to annealing.

**Table 2:** Nanofillers mixed with PP improved nanocomposites’ properties.

Methods for Obtaining Composite PP/CNT Materials	Investigated Properties	References
Ultrasonic irradiation method after extrusion of composite material	Slight oxidation of the polypropylene structure, decrease in molecular weight, increase in crystallinity, increase in modulus of elasticity, increase in thermal decomposition temperature, increase in volumetric resistivity, increase in thermal conductivity	[51]
The melting method using a twin-screw extruder followed by injection molding	Thermal conductivity, tensile modulus of elasticity, tensile strength and the stress at break are gradually increased, while the elongation at break is significantly reduced	[52]
The method of mixing a melt with a controlled time of CNT dispersion in a PP matrix.	Mechanical properties (increase in tensile strength and Young’s modulus)	[53]
The melting method using a twin-screw extruder followed by injection molding	Improvement in physical properties, conductive properties (the “percolation threshold” has been identified)	[56]
The method of additional treatment by microwaves after extrusion of composite material	Mechanical properties (an increase in mechanical strength and ultimate strength, while the nanocomposite exhibits elastoplastic properties)	[59]
Addition of an external nucleating agent (NA) based on sorbitol and control of the cooling rate.	Conductive properties (significant reduction in the percolation threshold)	[72]
Melt mixing technique with controlled residence time for CNT dispersion in a PP matrix using a twin-screw extruder	Rheological properties (lowest average molecular weight and lowest average molecular weight), electrical properties, mechanical properties (tensile strength and Young’s modulus increased linearly)	[74]
The melting method using a twin-screw extruder and the injection molding method; samples with the same MNT content, but with different mold temperatures and injection rates, were studied.	Electrical properties (an increase in mold temperature and injection rate leads to a decrease in electrical resistivity)	[76]
The method of shear impact	Mechanical properties (increase in hardness), electrical properties (dependence of electrical conductivity on shear and CNT content)	[77]
Preparation of a nanocomposite by compounding a melt using a twin-screw extruder	Detection of sensory properties, increase in impedance	[78]
The melting method using a twin-screw extruder followed by injection molding	Mechanical properties (improvement in tensile strength and modulus of elasticity), increased electrical conductivity, shielding efficiency	[80]

**Figure 9:**
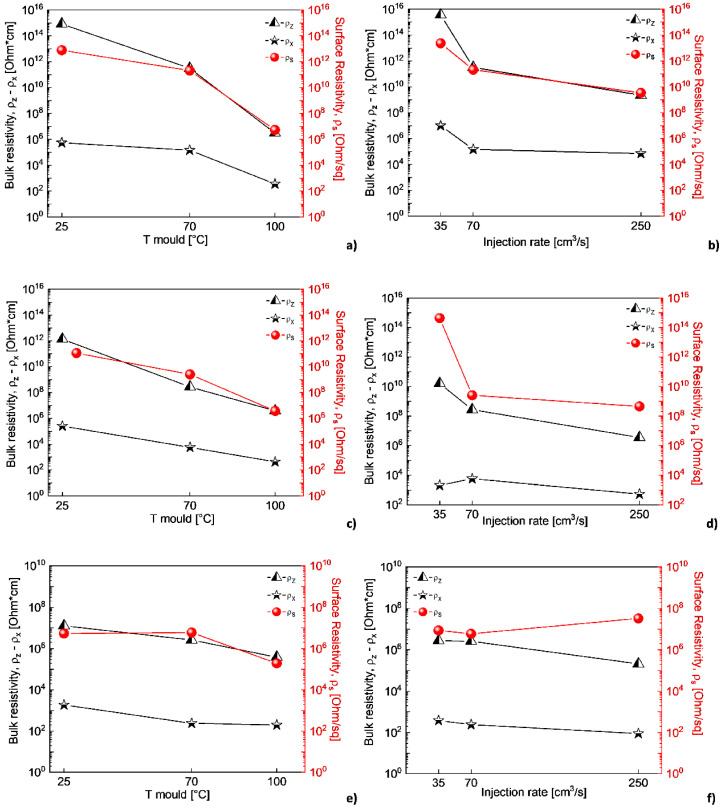
Effect of the mold temperature and injection rate on the electrical bulk and surface resistivity of (**a**) 2%-MWCNT nanocomposites, injection rate 70 cm^3^/s; (**b**) mold temperature 70 °C; (**c**) 3%-MWCNT nanocomposites, injection rate 70 cm^3^/s; (**d**) mold temperature 70 °C; (**e**) 4%-MWCNT nanocomposites, injection rate 70 cm^3^/s; (**f**) mold temperature 70 °C [83].

**Figure 10:**
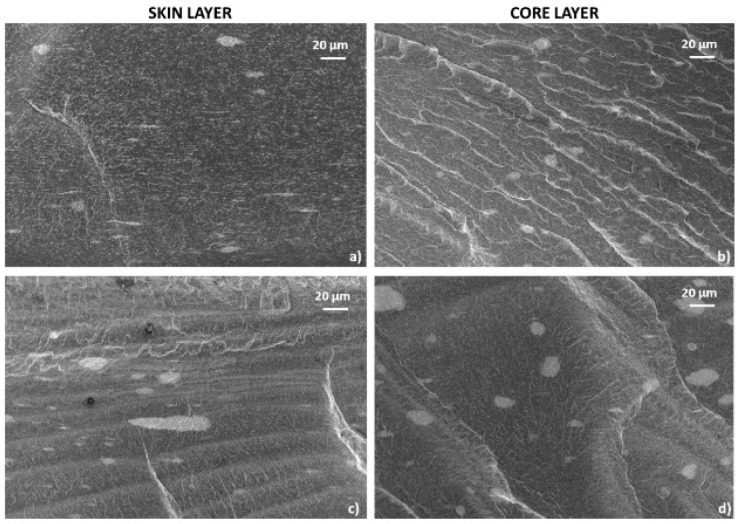
Enlargement of the FESEM images of the cross-section of 3%-MWCNT nanocomposite. The micrographs were obtained (**a**) from the skin layers of the nanocomposites manufactured at a mold temperature of 25 °C; (**b**) from the core regions of the nanocomposites manufactured at a mold temperature of 25 °C; (**c**) from the skin layers of the nanocomposites manufactured at a mold temperature of 100 °C; (**d**) from the core regions of the nanocomposites manufactured at a mold temperature of 100 °C.

In [82], the electrical and electromechanical characteristics (piezo-impedance) of PP/MWCNT composites were studied when exposed to alternating current (AC) and compared with their direct current counterparts (piezo resistance). The frequency characteristics and piezo-impedance were investigated taking into account two electrode configurations. The first configuration consisted of ordinary copper cables bonded with conductive paint (the so-called “resistive configuration”). The second option involved an increased dielectric constant, which was expected to strengthen the capacitive component (“dielectric configuration”). Based on the results obtained, it was concluded that it is possible to use the value of the phase angle of alternating current as a new parameter for quantifying the sensitivity of intelligent materials to deformations. This parameter provides not only higher sensitivity but also valuable information about resistive/capacitive properties without the need for a circuit model. The alternating current concept discussed here has proven to be a viable alternative for increasing the electromechanical sensitivity of carbon nanostructured nanocomposites. In addition to gaining new knowledge, these results contribute to the development of devices that are sensitive to deformation and motion, especially those based on flexible polymers, such as tactile sensors for human–machine interfaces, as well as for soft robotics. In [62], it was found that the electrical properties of PP/MWCNT nanocomposites transition from the insulating to the semiconductor range with an increase in the MWCNT content in the composite material. At the same time, the PP/MWCNT nanocomposite is not electrically conductive up to 3% by weight, whereas when the MWCNT content is higher than 3% by weight, due to the formation of a fully conductive grid, the nanocomposite behaves as a semiconductor. The electrical conductivity increases sharply from 10^−12^ S/m to 10^−1^ S/m, which is typical for the phenomenon of percolation. In addition, it was noted that the electrical conductivity of the PP/MWCNT nanocomposite does not depend on the injection molding temperature. The electrical properties of PP/CNT composite materials were also evaluated in [63], where a special melt mixing technique, described in the previous section, was used. As a result of the statistical theory of percolation, it was found that the threshold of electrical percolation is 1.4 wt.%, with a critical t value of 2.05. The volumetric electrical conductivity increases with an increase in the content of CNTs in composites. Thus, it was concluded that due to the controlled holding time and excellent dispersion condition, improved electrical conductivity in the polypropylene matrix was achieved through a new melt mixing process, without additional modifications. Rheological, morphological, and electrical measurements confirmed that an optimized dispersion and electro-flowing mesh of SWCNTs was formed, containing about 1.28% by weight of SWCNTs in polypropylene. However, taking into account the fact that CNTs have better properties compared to CNTs, the threshold of electrical percolation is at 1.28 wt.%, which may not meet expectations. There are also studies where certain conditions for the production of nanocomposites are used to impart conductive properties to a polymer material simultaneously with the introduction of carbon nanotubes [81,83,84]. In [83], the effect of injection molding conditions on the crystal structure and electrical resistance of PP/CNT nanocomposites was investigated. Composite materials were obtained by mixing a melt in a twin-screw extruder with simultaneous rotation, and then composite materials based on polypropylene with MWCNTs were obtained by injection molding. Samples with the same content of MWCNTs, but with different temperatures of the injection mold and velocity, were obtained. The effect of mold temperature and injection velocity on both the volumetric resistivity (ρ_z_ and ρ_x_) and the surface resistivity (ρ_s_) of formulations based on 2%, 3% and 4% MWCNTs is shown in the figure. Electrical characteristics showed that an increase in mold temperature and injection rate leads to a decrease in electrical resistivity (Figure 9). Optical and field emission scanning electron (FESEM) microscopies were used to observe the morphology of the MWCNT clusters (Figure 10). The authors found that adjusting the injection molding processing conditions leads to a modification of the crystalline structure together with the inter-cluster connections, which results in tunable electrical properties. The SEM analysis showed morphological differences in the samples produced at temperatures of 25 °C and 100 °C. In the core area, many rounded clusters are visible, which gradually become more elongated in the direction of the plane, passing into the shell layers. The authors suggested that these morphological differences in the samples do not fully justify the strong influence on electrical properties.

**Figure 11:**
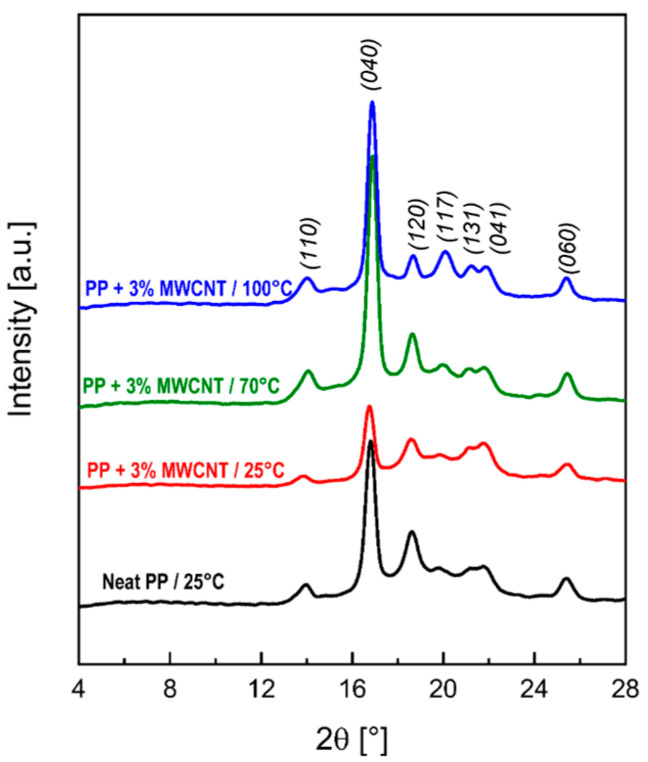
X-ray diffraction patterns of neat PP in comparison with the 3%-MWCNT nanocomposites manufactured at the three different mold temperatures [83].

The authors refer to the fact that PP can crystallize in three polymorphic crystallographic forms (monoclinic α-phase, hexagonal β-phase and triclinic γ-phase), each of which has its own characteristic peaks on radiographs. It was found that the PP/CNT composite with a 3% CNT content belongs to the γ-phase (2θ = 20.1°) (Figure 11).

The decrease in the electrical resistivity can be associated with the presence of a larger fraction of a crystalline γ-phase, which seems to favor a more efficient inter-connection of MWCNT clusters, rather than a significant modification of the intra-cluster morphology.

In [84], the authors investigated the simultaneous effect of the presence of carbon nanotubes (CNTs) and shear effects on rheological and electrical properties, as well as on the crystallization behavior of nanocomposites based on polypropylene (PP). The results of rheological analyses showed that with an increase in the CNT content in the polymer matrix, the hardness increases, and the maximum hardness value is reached at a CNT content of 2% by weight. The results of isothermal crystallization showed that an increase in the CNT content in the polymer also affects the crystallization temperature. At the same time, it was found that the crystallization temperature does not change during the shift. In non-isothermal crystallization, the kinetics of crystallization improve with an increase in the CNT content, and this becomes more pronounced with shear. The results of the thermal analysis confirmed that the melting point decreased slightly, and the crystallinity remained almost unchanged with an increase in the CNT content to 2%. However, when the CNT content increased to 4%, the crystallinity decreased significantly due to limited crystallization. When this nanocomposite was sheared, its crystallinity increased. It was also found that electrical conductivity increases with increasing CNT content, while it decreases with shear, and the vulnerability of nanocomposites to shear decreases with increasing CNT content.

The unique conductive mechanical and electrical properties of CNTs make it possible to use these nanoparticles as nano-additives in a polymer matrix to create strain sensors. In [85], polypropylene/carbon nanotube (PP/CNT) nanocomposites with different concentrations of CNT (i.e., 1, 2, 3, 5 and 7 wt.%) were prepared. PP/CNT composites were obtained by compounding the melt using a twin-screw extruder. Composites were tested as load cells for structural control. Such sensors were embedded in cement mortar prisms and tested in a 3-point bending mode, registering a change in impedance with increasing load. It has been established that PP/CNT nanocomposites with 5 and 7 wt.% CNT have interesting sensory properties. In particular, the best result was observed for the PP/CNT nanocomposite with 5 wt.% CNT. The melting point of PP/CNT nanocomposites decreases slightly compared to pure PP (by about 2 °C) due to a decrease in the size of the crystallites. It has also been established that CNTs significantly affect mechanical properties, in particular, the modulus of elasticity and deformation during fracture. The modulus of elasticity increased slightly at 1 wt.% of the CNT content (+2%), while a more significant increase was recorded at higher CNT loads (+11%, 15% and 16% with the addition of 2, 3 and 5 wt.%, respectively). With a further increase in the concentration of CNTs, the modulus of elasticity begins to decrease. The strain-sensitive ability of PP/CNT nanocomposites embedded in cement mortar was also studied when measuring impedance changes. The CNT content of 1 and 2% in the polymer matrix did not show significant results. However, the CNT content of 3.5 and 7% yielded good results. The impedance began to increase at very low loads (350–450N) and acquired an initial value when the load was removed. When measuring the average gauge factor (GF), the best result was observed for a PP/CNT nanocomposite with 5 wt.% CNT. It was about 1400.

Based on the results obtained, the authors declare the possibility of creating new cheap strain gauges.

### 2.5. The Use of CNTs in a PP Matrix to Create Composite Materials for Electromagnetic Shielding

As noted earlier, CNTs have outstanding electrical properties, which makes them ideal for use as fillers in a polymer matrix. A composite material based on polymer and CNTs can provide effective shielding from electromagnetic radiation due to the unique properties of carbon nanotubes. The addition of CNTs to the polymer matrix opens up new possibilities for creating composite materials with improved electromagnetic shielding properties. This can find applications in various fields, including electrical engineering, telecommunications and the defense industry.

Composite materials based on polypropylene doped with nanoparticles, namely graphene nanoplastics (GNPs) and multi-walled carbon nanotubes, were obtained in [86]. Two types of graphene nanoplastics from different manufacturers were used in the study: GNPS (EMFUTUR Technologies Ltd., Villarreal, Spain) and GNPV (MegaLab Ltd. Larnaca, Cyprus). Samples were made in the form of panels with a thickness of 1 mm, and the properties of the obtained composites were studied for the possibility of shielding electromagnetic interference (EMI).

**Figure 12:**
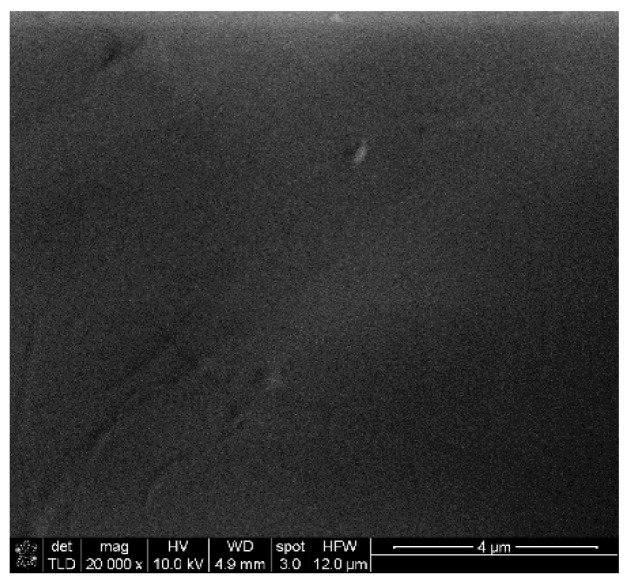
SEM characterization (×20,000, Au coated) of pure PP surface.

**Figure 13:**
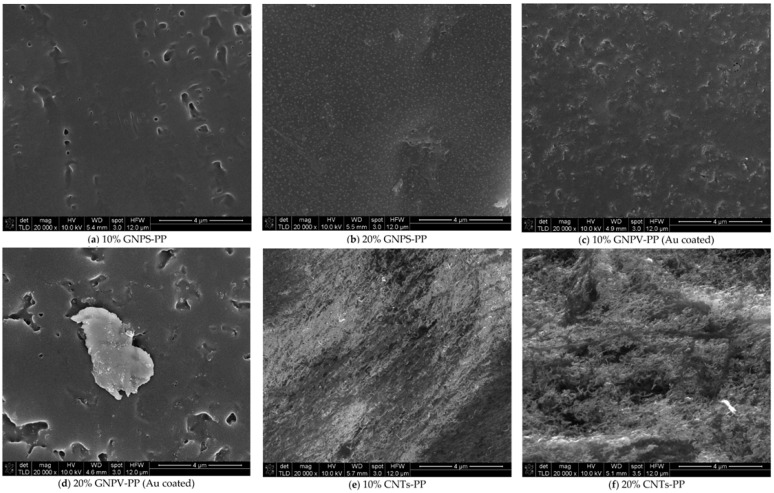
SEM characterization (×20,000 magnification) of composite materials: (**a**) 10% GNPS-PP, (**b**) 20% GNPS-PP, (**c**) 10% GNPV-PP, (**d**) 20% GNPV-PP, (**e**) 10% CNTs-PP, (**f**) 20% CNTs-PP.

The ingredients were carefully weighed and mixed using a roller to obtain a homogeneous composition with suitable properties. Roller is the most common equipment for batch mixing. To obtain the most homogeneous composition, all parameters of the mixing process (temperature, relative roll speeds or clearance) must be optimized for specific materials. Accordingly, various tests were carried out to achieve optimal conditions for processing polypropylene granules. It was found that 170–180 °C is the appropriate temperature range to achieve the desired mechanical properties when mixing a polymer with nanomaterials in powder form. Various preliminary processing tests were carried out on a hot rolling mill to achieve the necessary mechanical properties of the hot soft composite, such as tear strength and tensile strength, so that the composite material can be removed from the hot rolls in the form of a 1 mm thick sheet. The composition was consistently adjusted until the mixtures were suitable for removal. The authors state that the surface features of nanocomposite materials (boundaries, voids and other inhomogeneities) are important due to the possibility of using these materials to protect against electromagnetic interference. Additives can act as static charge accumulation sites or disrupt the electron flow. To study the distribution of nanofillers in the studied composites, the samples were examined using SEM (Figure 12 and Figure 13). The authors found that voids and a porous structure are visible on the surface of the composite even at 10% concentrations of NPV and GNPS. In the case of a PP/CNT composite with a content of 10% CNT, oriented CNTs (in the diagonal direction) are observed. At 20% CNT, agglomerations are visible, which leads to a loss of any orientation in the composite sheet, and a random distribution over the surface is observed.

**Figure 14:**
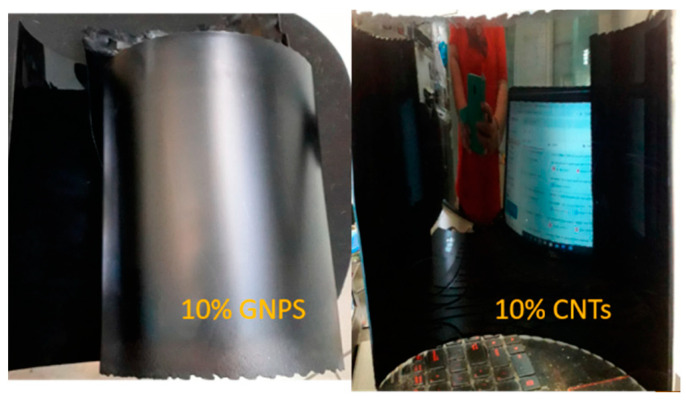
Examples of composite material panels [86].

**Figure 15:**
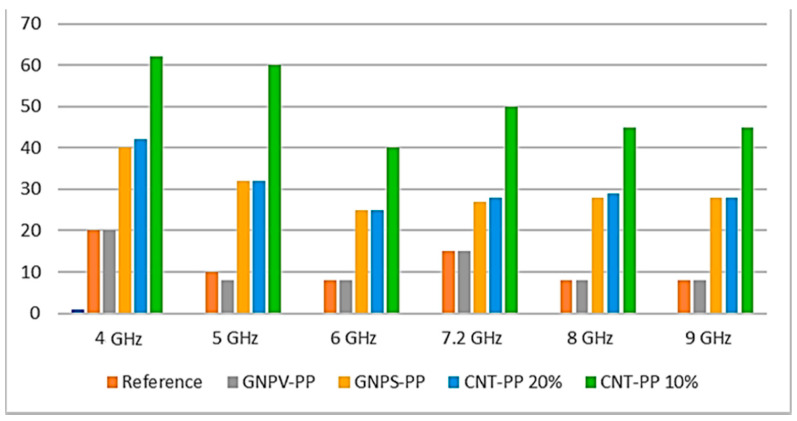
Graphical representation of the various materials’ attenuation efficiency [86].

Structural and morphological studies have shown that the obtained composite materials have certain properties, and the concentration and nature of the filler affect the structure of nanomaterials and their conductivity. In the case of using GNP as nano-additives, composites with lower conductivity and less effective shielding of electromagnetic interference are obtained. As for CNT-PP composite panels, they were found to exhibit excellent EMI attenuation of over 40 dB at a CNT concentration of 10%. The authors emphasize the importance of this development, as PP is one of the most widely used polymers, the best for injection molding and almost infinitely recyclable (Figure 14 and Figure 15).

Ashish and Vishal, in their works [87,88], proposed a method for creating a nanocomposite material for use as a mechanically durable shielding material to meet industrial shielding needs. In [12], a technology for producing MWCNT/PP nanocomposites using a twin-screw extruder is described. Studies have shown a low percolation threshold (0.1% by weight), which indicates an excellent dispersion of MWCNT in a polypropylene matrix with a DC conductivity of 4.21 × 10^−6^ S/cm. Nanocomposites exhibit increased tensile strength and modulus of elasticity, which reaches 33.24 and 611.15 MPa at 5 wt.% MWCNT. Due to the improvement of these properties, the authors conclude that it is possible to use these composite materials as mechanically durable protective materials to protect against electromagnetic interference in a wide frequency range (to be used the nanocomposite materials as mechanically durable shield materials for protection against EMI in a wide frequency range). Later, Ashish and Vishal [88] investigated the mechanical properties of multi-walled carbon nanotubes (MWCNTs) and carbon fiber-reinforced polypropylene nanocomposites, direct current conductivity and electromagnetic interference EMI properties. The nanocomposites were obtained by melting using a twin-screw extruder, followed by injection molding. Studies have shown an improvement in the tensile strength (by 31.14%) and modulus of elasticity of nanocomposites (60.14%). At the same time, the DC electrical conductivity improved from 2.07 × 10^−10^ to 9.58 × 10^−2^ S/cm. The nanocomposites demonstrated a 51.9 dB shielding efficiency at maximum filler loading in the X-band (8.2–12.4 GHz) for a 2 mm thick sample.

## 3. Theoretical Studies on Polymer Composite Materials Based on Polypropylene and Carbon Nanotubes

**Figure 16:**
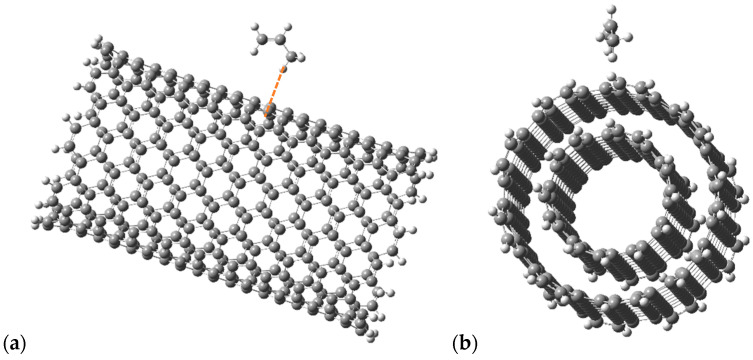
Variants of the orientation of the monomer relative to the CNT, which are considered in the study using the example of (**a**) single-walled CNTs and (**b**) double-walled CNTs.

**Figure 17:**
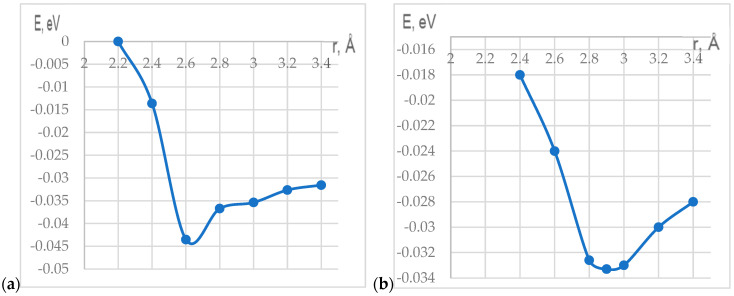
Energy curves of the interaction between the C_2_H_4_O monomer and (**a**) SWCNT (9, 9); (**b**) MWCNT (6, 6) and (9, 9).

In study [89], it was noted that the main mechanism for creating polymer composite materials by doping CNTs is the adsorption interaction between the components of the complex. To clarify the applicability of these results to the PP nanocomposite, we investigated the interaction process between CNTs and PP monomers. The density functional theory was applied to study the structural features and electron-energy structure of a PP-based nanocomposite doped with carbon nanotubes, as well as to study the mechanisms of interaction between CNTs and polypropylene fragments [90]. The essence of this method is to be used in the description of atomic and molecular electron density distribution systems. A hybrid approximation method, namely the B3LYP method, was chosen to study the system. The main advantage of the B3LYP method is its high accuracy. The calculations were carried out using a valence-split 3–21G-type basic set. This functionality with the selected basic set is well adapted to the selected systems. Theoretical studies on the interaction between the structural unit of the nanomaterial, polypropylene (PP), and the surface of single- and double-walled CNTs were performed using quantum chemical modeling and the DFT calculation method in Gaussian 09W software [91]. A model of a CNT molecular cluster with boundary pseudoatoms was used. This model allows for the identification of a specific section of the extended nanotube system and performs the necessary calculations specifically for this purpose. The cluster length was chosen so as to avoid the influence of edge effects on the process. Pseudoatoms were used to compensate for broken chemical bonds at the cluster boundary. In the case under consideration, hydrogen atoms, playing the role of pseudoatoms, are well suited for this role. We calculated the energy characteristics of the adsorption processes of a single monomeric unit (C_3_H_6_) of polypropylene on single-walled achiral CNTs of the “arm chair” type with chirality indices (9.9) and double-walled achiral CNTs of the “arm chair” type with chirality indices (6.6) for the inner CNTs and (9.9) for the outer CNTs. In the course of quantum chemical research, maps of the electrostatic potential, known as maps of the electrostatic potential energy or surfaces of the molecular electric potential, have also been analyzed, illustrating the three-dimensional charge distribution of molecules. By studying the distribution of the electrostatic potential and analyzing natural binding orbitals (NBOs), active centers responsible for intermolecular interaction were identified. The active center in the polypropylene molecule turned out to be a hydrogen atom. The process of adsorption of the C_3_H_6_ polypropylene fragment to the central part of the CNT cluster was simulated by stepwise approximation of the molecule by the active center of the hydrogen atom to the selected carbon atom of the nanotube perpendicular to the surface of the CNTs (the line of movement of the monomer is perpendicular to the longitudinal axis of the nanotube) (Figure 16). As a result of the calculations performed, the energy values of the systems were obtained at each step, which made it possible to plot curves of the dependence of the interaction energy on the distance between the selected monomer and the CNT (Figure 17).

It is established that each curve has a minimum corresponding to the interaction at certain distances.

The adsorption energy was calculated as the difference between the total energies of the adsorption complex and the sum of the energies of the non-interacting CNTs and the monomer under study (C_3_H_6_):

∆E_a_ = E_ad.com_. − (E_CNT_ + E_mol_) (1)

where E_ad.com._ represents the energy of the adsorption complex obtained as a result of calculations, E_CNT_ is the energy of pure CNT, E_M-ЛbI_ is the energy of C_3_H_6_.

The values of ∆Ea indicated physical interaction (adsorption) between the C_3_H_6_ monomer and the CNT cluster (Table 1):

(1) The energy value during the interaction between C_3_H_6_ and a SWCNT turned out to be 1.60 MeV at an adsorption distance of Rad = 2.6 Å;

(2) The value of the adsorption energy during the interaction between C_3_H_6_ and MWCNT was 3.33 MeV at a distance of Rad = 2.9 Å.

Table 3 shows the main results obtained during the study of the adsorption interaction of the monomer P with the outer surface of SWCNT and MWCNT.

Therefore, the established fact of the interaction between the structural unit of the PP polymer and the surface of achiral single- and double-walled carbon nanotubes makes it possible to predict the possibility of creating stable complexes in composite polymer material based on polypropylene reinforced with nanotubes.

## 4. Conclusions

The analysis of the works devoted to theoretical and experimental studies of polymer composites based on polypropylene modified with the addition of nanomaterials (nanoparticles and carbon nanotubes) demonstrates the scale of application of these composite materials. The applications cover a wide range of fields, including materials science and nanotechnology.

As modern works show, the PP/CNT composite material is of great interest for further study and use. The development of its production technologies contributes to the creation of new physical objects with both scientific and applied value.

Current research is aimed at finding new modifying additives, as well as a way to modify them into a polymer matrix that will improve the characteristics of a polymer material based on PP. This review highlights not only the unique physico-chemical properties of polypropylene, but also the effects that occur when PP is modified with nanofillers.

The composites created in this way have improved mechanical, electrical, and radio-absorbing properties, opening prospects for their use in various industries.

## Figures and Tables

**Table 1:** Nanofillers mixed with PP, improving nanocomposites’ properties.

Nanofiller	Improved Properties	References
TiN	Mechanical (tensile strength, bending, impact strength, microhardness)	[30]
Si_3_N_4_	Mechanical (tensile modulus of elasticity, bending strength)	[33]
Si	Rheological (flow rate)	[34]
CaCO_3_	Mechanical (hardness and impact strength), crystallinity, thermal conductivity	[35]
Carbon nanofiber (CNF)	Mechanical (flexural strength) and thermal	[39]
Nanoclay (NC)	Mechanical (tensile strength, Young’s modulus), thermal and rheological properties	[41]
Graphene	Mechanical (tensile strength, modulus of elasticity) and electrical conductivity	[41]
Vegetable fibers (coconut, oil palm, and corn plant fibers)	Mechanical (the exception of flexural strength), thermal and fire-resistant characteristics	[42]
Nanosilica (NS)	Mechanical characteristics (Young’s modulus, tensile strength)	[43]

**Table 3:** The results of the adsorption interaction between the PP monomer and the outer surface of SWCNT and MWCNT.

Types of CNT	Active Center	Adsorption Distance, R_ad_, Å	Adsorption Energy, ∆E_a_, meV
SWCNT	H	2.6	1.6
MWCNT	H	2.9	3.33
